# Simultaneous measurement of formic acid, methanol and ethanol in vitreous and blood samples of postmortem by headspace GC-FID

**DOI:** 10.1186/s12995-017-0184-3

**Published:** 2018-01-08

**Authors:** Hamideh Ghorbani, Alireza Nezami, Behjat Sheikholeslami, Arya Hedjazi, Mahnaz Ahmadimanesh

**Affiliations:** 1 Legal Medicine Research Center, Legal Medicine Organization, Tehran, Iran; 2 Department of Pharmacodynamics & Toxicology, Mashhad University of Medical Science, School of Pharmacy, P. O. Box: 91388-13944, Mashhad, Iran; 3 Faculty of Pharmacy, Lorestan University of Medical Sciences, Khorramabad, Iran

**Keywords:** Methanol, Formic acid, Methyl formate, Postmortem, Poisoning, Measurement

## Abstract

**Background:**

Formic acid (formate) is the main reason for toxicity and death through methanol poisoning. The simultaneous determination of methanol, ethanol, and formate in the body can help to discover the cause of death and is useful in the diagnosis of acute methanol poisoning. The measurement of formate is not yet available in Iran. With regard to the increasing rate of methanol poisoning and its related mortality in Iran, as well as the main role of formate in methanol poisoning, this study was designed to set up an analytical method for the concurrent determination of ethanol, methanol, and formate.

**Methods:**

Following the modification of a previously developed gas chromatography method, vitreous and blood samples of 43 postmortem cases with a history of methanol intoxication were collected over a period of 2 years at the Legal Medicine Organization of Mashhad. Thereafter, ethanol, methanol, and formate concentrations were measured by headspace GC/FID. Formate esterification was performed by the methylation of formate with sulfuric acid and methanol. In order to confirm the esterification method for the production of methyl formate, we used gas chromatography with a mass detector (GC/MS) because of its higher sensitivity and accuracy. Furthermore, the correlations between formate and methanol concentrations in blood and vitreous samples, and between formate and methanol were investigated.

**Results:**

A significant relationship was found only between methanol concentrations in blood and vitreous samples (*P* < 0.03).

**Conclusions:**

In postmortems, with the passage of time since alcohol ingestion, the measurement of only methanol concentration cannot determine the degree of toxicity or the cause of death. Therefore, using the present analytical method and measurement of formic acid, we can estimate the degree of toxicity and cause of death.

## Background

Methanol (wood spirits) is a widely used commercial and industrial alcohol. It is also found in the production process of non-standard alcoholic beverages using grape twigs [1]. In the human body, it is first metabolized to formaldehyde by alcohol dehydrogenase in the liver. Then it is converted to formate, which, in turn, is transformed to CO_2_ and H_2_O [1–3]. Methanol itself has low toxicity, and there is a relatively negligible correlation between toxicity or mortality and methanol blood or serum concentrations [4, 5]. Formaldehyde is very toxic but it has a short half-life and does not accumulate; hence, no considerable formaldehyde concentrations are found in body fluids and tissues following the administration of methanol [6]. Formic acid is considered to be the main toxic compound produced in the course of methyl alcohol metabolism. Formic acid accumulates in the body and is the main reason for toxic effects and death through methanol poisoning [6–8]. Commonly reported adverse effects related to high serum concentrations of formic acid include visual damage, optical nerve injury, abdominal problems, nausea, and headache. Subsequently, high formic acid concentrations result in respiratory problems, renal failure, and finally lead to coma and death [9]. Therefore, it seems necessary to determine the serum concentrations of methanol and especially of formic acid, in order to evaluate the causes of these observed adverse effects.

The measurement of formate is not yet available in Iran. With regard to the increasing rate of methanol poisoning and its related mortality in Iran, and also to the role of formate in methanol poisoning and the importance of its measurement in determining the causes of poisoning and death [10], this study was designed to set up an analytical method for the detection of formic acid in human samples. While flame ionization detectors (FIDs) are commonly used to measure methyl and ethyl alcohol, they are almost unable to determine formate concentration [9, 11, 12]. An esterification step is needed for formic acid detection [11, 12]. Enzymatic methods have also been used to determine the formate concentration [13]. Headspace gas chromatography (HSGC) has been utilized for the detection of formic acid using sodium propionate as an internal standard [14]. Also, the GC/MS has recently been used for the detection of formic acid [15, 16].

In the present study, using a previously developed gas chromatography method with some modification [11], we simultaneously measured formic acid, methanol, and ethanol concentrations in 43 postmortems of vitreous and blood samples by headspace GC/FID. Furthermore, we investigated the relationship between concentrations of analytes in blood and vitreous samples.

## Methods

Methyl formate, formic acid (95%), sulfuric acid (97%), ethanol (96%), methanol, acetonitrile (99.9%), dichloromethane, and distilled water were purchased from Merck Company (analytical grade).

### Sample collection and storage

In the present study, 43 victims who died of methanol poisoning were investigated from April 2014 to April 2016. This work was approved by Tehran Legal Medicine Research Center Ethics Committee. All bodies were transferred to the Mashhad Legal Medicine Organization (LMO) within 24 h of death. For all cases, the interval of time between death and sampling was less than 24 h. Also, the interval of time between sampling and the analyses of the samples was less than 2 h. All autopsies of major organs were studied together with a review of clinical histories. Blood and vitreous samples were analyzed to determine methanol, formic acid, and ethanol concentrations. Blood samples were taken from the femoral region and stored in tubes containing sodium fluoride (NaF). All the blood and vitreous (without preservatives) samples were stored at −4 °C. Moral consent was taken from the family of the deceased before the experiment.

Headspace GC/FID was used in the sample analysis for the simultaneous determination of ethanol, methanol, and formic acid. The GC/MS was also used to confirm the results of GC/FID. The detailed conditions of these devices were listed as follows:

### GC/FID headspace conditions

The 7890A Headspace GC/FID model was provided by Agilent. Data analysis was performed using Agilent chemstation REV.B.04.03. Nitrogen carrier gas (purity 99.9992%) was supplied by nitrogen cylinders. Hydrogen gas was supplied from a Chebios hydrogen generator. The complete details of the GC/FID procedure are presented in Table 1.

**Table 1:** Parameter values for HS GC/FID

Parameter	Type	Value
Carrier gas	Nitrogen	Nitrogen cylinders
Column:	Capillary column of the Restek Corporation (J & W RTX-BAC1 Model)	Dimensions = 30 m * 530 μm * 3 μm Flow = 1.5 ml/min Pressure = 1.2 psi
Detector:	FID	Heater = 250 °C H_2_ Flow = 40 ml/min Air Flow = 400 ml/min
Oven:	Gradient	Initial temperature = 60 °C Hold time = 5 min Rate = 10 °C Final temperature = 130 °C Run time = 12 min
Inlet:		Temperature = 90 °C Total flow = 79.5 ml/min Septum Purge Flow = 3 ml/min Gas Saver = on
Injector	Headspace split (1:20)	200 °C
Headspace:		Zone temperature = 40 °C Event time = 15 min

### GC/MS conditions

The GC/MS was from the Agilent Company (6890 N-5978B). Helium carrier gas was supplied by helium cylinders. The scan mode was used for the GC/MS analysis. Data analysis was performed using MSD chemstation G1701EA E.02.01.1177. The full details of the GC/MS are shown in Table 2.

**Table 2:** Parameter values for GC/MS

Parameter	Type	Value
Carrier gas	Helium	Helium cylinders
Column:	capillary column (19,091 F) - General	Size 5 mm Flow = 0.5 ml/min
Detector:	Mass	
Oven:	Gradient	Initial temperature = 65 °C Rate = 5 °C Final temperature = 90 °C Run time = 7.5 min
Inlet:		Temperature = 90 °C Total flow = 79.5 ml/min Septum Purge Flow = 3 ml/min Gas Saver = on
Injector	split less	90 °C

### Derivatization

Since gas chromatography cannot detect formic acid, it is necessary to convert it to methyl formate by esterification (derivatization) before its quantification in blood and vitreous samples. The following steps were performed for the derivatization of formic acid: 1 mL samples containing formic acid were transferred to headspace vials (Restek) coated with a microfilm; 500 μl of concentrated sulfuric acid (97%) was added as a catalyst and then mixed; the samples were incubated for 20 min at room temperature; 30 μl of pure methanol (99.9%) for the conversion of formic acid to methyl formate and 30 μl acetonitrile (0.197 M was prepared from pure acetonitrile (99.99%) by diluting with water) as an internal standard were added; the resultant was shaken; and finally, following a 20-min incubation at room temperature, the samples were prepared for injection [11, 12].

### Preparation of stock solutions and calibration curve

At first, pure methyl formate was injected into the GC/MS to ensure that detection by GC/FID and the determined retention time were properly conducted [15, 16]. Thereafter, standard samples of methyl formate were prepared from formic acid according to the literature [9, 11, 12]. After this, liquid–liquid extraction was done using a mixture of water and di-chloromethane (1:1, *v*/v), and organic layers were infused to the device. Figures of pure methyl formate were compared with those produced from formic acid in samples, in order to ensure the accuracy of the derivatization process.

In the next step, separate standard aqueous samples of ethanol, methanol, and formic acid were prepared and then, after the derivatization of formic acid using the procedure mentioned above, injected to HSGC/FID to define their retention times. To draw the calibration curve, 10 concentrations of ethanol, methanol, and formic acid were prepared in concentration ranges of 20 to 200 mg/dL, 50 to 500 mg/dL, and 0.5 to 150 mg/dL, respectively. All bioanalytical method validation procedures for calculating accuracy, precision, recovery, and limit of quantitation (LOQ) were done according to the Food and Drug Administration (FDA) guidance for industry [17].

### Application of method in biologic matrices

**Fig. 1:**
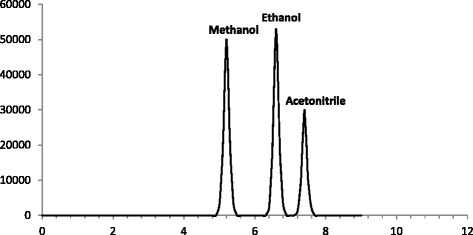
HSGC/FID chromatogram of blank human vitreous spiked with methanol (200 mg/dl), ethanol (150 mg/dl) and acetonitrile (0.197 M) as an internal standard

**Fig. 2:**
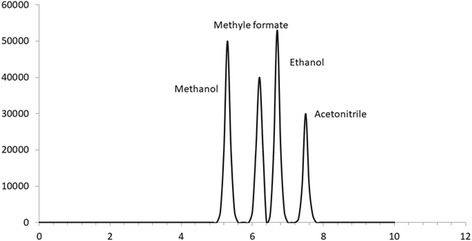
HSGC/FID chromatogram of blank human vitreous spiked with methanol (200 mg/dl), methyl formate (65 mg/dl), ethanol (150 mg/dl) and acetonitrile (0.197 M) as an internal standard

**Fig. 3:**
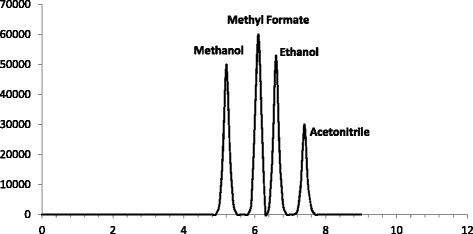
HSGC/FID chromatogram of blank human vitreous spiked with methanol (200 mg/dl), methyl formate (65 mg/dl plus 20 μl of pure methyl formate), ethanol (150 mg/dl) and acetonitrile (0.197 M) as an internal standard

The blank samples (blood and vitreous) were mixed with a certain amount of ethanol and methanol and injected into the device (Fig. 1). In the second step, they were mixed with a certain amount of formic acid and injected into the device following derivatization to methyl formate (Fig. 2). In order to confirm the retention time of methyl formate, 20 μl of pure methyl formate was added to the samples and the samples were spiked (Fig. 3). This work was performed three times in 3 days and the mean values were compared with the desired values. Since ethanol and methanol may affect the process of derivatization, we analyzed methanol and ethanol concentrations in all samples before derivatization.

### Pearson correlation

Relationships between the blood and vitreous concentrations of methanol and formic acid were evaluated using the Pearson correlation tool.

### Statistical analysis

Statistical analysis was performed using SPSS version 22. Appropriate parametric tests were used. Two tailed tests with a *P* value less than 0.05 were considered statistically significant.

## Results

### Analytical method

The retention time for methyl formate in the GC/MS was 1.85 min. The retention time in GC/FID for methyl formate, methanol, and ethanol were 6.2, 5.3, and 6.7 min, respectively.

The calibration curves for all analytes were made in a previously mentioned range of concentrations. The correlation coefficients (R2) for formic acid, methanol, and ethanol were 0.992, 0.999, and 0.999, respectively. In this study, a recovery rate of over 89% was calculated. For all analytes involving the blood and vitreous samples, the limit of detection (LOD) and the LOQ were 0.5 mg/dL and 1.5 mg/dL, respectively. The mentioned bioanalytical method provided an accuracy of 97.3–102.4%, and the precision, relative standard deviation (RSD %) was better than 9.6% for control samples (Table 3).

**Table 3:** Determination of accuracy and precision for formic acid, methanol and ethanol in human vitreous and whole blood

Concentration (mg/dl)	Within-day (n = 3)			Between-day (*n* = 3)		
	Mean ± SD	Accuracy	Precision	Mean ± SD	Accuracy	Precision
Formica acid (blood)						
0.5	0.49 ± 0.02	98.0	4.08	0.49 ± 0.01	99.3	2.32
75	75.6 ± 1.5	100.8	2.01	73 ± 1	97.3	1.37
150	153.6 ± 6.1	102.4	3.97	153.6 ± 7.7	102.4	5.01
Methanol (blood)						
50	54.2 ± 4.7	101.8	3.75	48.8 ± 1.04	97.6	2.13
250	248.6 ± 7.6	99.4	3.09	247.5 ± 8.4	99.1	3.41
500	491.6 ± 47.2	98.3	9.61	503.3 ± 35.4	100.6	7.04
Ethanol (blood)						
20	20.4 ± 0.9	102.0	4.67	20.2 ± 0.8	101.2	4.12
100	97.5 ± 7.6	97.5	7.89	98.8 ± 9.2	98.8	9.3
200	199.3 ± 18.1	99.6	9.10	203.3 ± 16.6	101.6	8.19
Formica acid (vitreous)						
0.5	0.5 ± 0.01	101.1	3.34	0.5 ± 0.01	100.8	3.37
75	76.5 ± 0.7	102.0	0.98	76.1 ± 3.5	101.5	4.69
150	148.7 ± 4.1	99.2	2.77	150.5 ± 4.8	100.3	3.20
Methanol (vitreous)						
50	49.2 ± 1.6	98.5	3.32	50.3 ± 2.7	100.7	5.47
250	251.2 ± 14.4	100.5	5.73	255.8 ± 12.3	102.3	4.82
500	499.1 ± 35.5	99.8	7.12	506.6 ± 33.2	101.4	6.57
Ethanol (vitreous)						
20	19.5 ± 0.4	97.7	2.13	19.8 ± 0.7	98.9	2.87
100	99.5 ± 5.07	99.5	5.09	100.2 ± 4.36	100.2	4.36
200	204.3 ± 12.6	102.2	6.19	200.6 ± 11.01	100.3	5.48

### Demographic data

The age range of the deceased persons was 23–81 years, with the average being 42.11 years (six females and 37 males). The dose and the time of ingestion, and the history of consumption were unknown. Therefore, the time interval between ingestion and death was unclear, and it was not possible to find a correlation between dosage, ingestion time, and incidence of death.

### Clinical finding

**Fig. 4:**
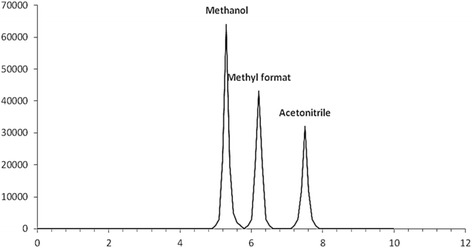
HSGC/FID chromatogram of a human vitreous sample of post-mortem containing, methanol (310.5 mg/dl), methyl formate (70.08 mg/dl) and acetonitrile (0.197 M) as an internal standard

Measured concentrations in both blood and vitreous samples are shown in Table 4. Also, the minimum and maximum concentrations for ethanol, methanol, and formic acid in the blood and vitreous samples are shown in Table 4. The mean and median values of concentrations, and correlations between mean concentrations were analyzed. A real sample chromatogram is displayed in Fig. 4.

**Table 4:** Mean and median of methanol, ethanol and formic acid concentrations in vitreous and blood

Analytes	Methanol mg/dL		Formic acid mg/dL		Ethanol mg/dL
Matrix	Vitreous	Blood	Vitreous	Blood	Vitreous
Mean ± SD	162.3 ± 179.5	131.9 ± 146.5	68.7 ± 37.5	79.6 ± 39.5	21.5 ± 48.2
Median (Min-Max)	80 (0–591)	59 (0–507.3)	73 (0–132.8)	84 (0–157)	1.2 (0–227.5)

In some cases, where methanol concentrations were under the limit of detection, formic acid concentrations were detected and vice versa. Considering the values of the Pearson correlation coefficient for methanol and formic acid, it appears that there is a very strong linear relation between the blood and vitreous concentrations of methanol (correlation coefficient: 0.987; significance: 0.00). However, no significant correlations were found between concentrations of formate in the blood and vitreous samples. Moreover, formate and methanol concentrations in both the blood and vitreous samples showed weak correlations (correlation coefficient: < 0.2).

## Discussion

The incidence of methanol poisoning and related mortality has increased worldwide in the last decade [10]. Also, owing to the consumption of contaminated beverages, its rate has increased in Iran [10]. In a methanol-poisoning outbreak in Mashhad in May 2009, 11 out of 25 patients referred to Imam Reza Hospital died [10].

With regard to the prohibition of alcohol consumption in Iran, alcoholic beverages are usually produced using illegal and non-standard (contaminated) methods [10]. In most cases, due to the existence of wood during fermentation, methanol concentration is considerable in the final product. Since handmade beverages have low concentrations of ethanol, consumers usually drink large amounts of liquor to reach the desired mood and effective blood concentrations. Therefore, the amount of methanol intake increases. This can be associated with an increased risk of poisoning. As a matter of fact, the incidence of deliberate poisoning or suicide by methanol is very low in Iran; methanol poisoning is generally accidental and unintentional [10, 18]. In unintentional poisoning, especially in a case of contaminated beverage consumption, simultaneous determination of methanol, ethanol, and formic acid in the body can help to discover the cause of death; it is also useful in the diagnosis of acute methanol poisoning [19–22]. Nowadays, GC/FID is an appropriate quantifying device available in most laboratories [9, 11, 12].

Given that the GC/FID device is often used for alcohol detection at the LMO in Iran, this study could provide a suitable method for the simultaneous determination of alcohol and formic acid. In this study, we also used the GC/MS to guarantee the sensitivity and accuracy of the mentioned GC/FID method since it is more sensitive and accurate than GC/FID [23, 24]. Compared with the GC/FID method developed by Bursova et al. [9], the present method provided smaller values for LOD and LOQ. Also, Bursova et al. [9], utilized two different columns in the GC/FID to validate the method, but we used two different chromatographic devices, GC/MS and GC/FID. We used the general column of the GC/MS (used to identify all materials) without headspace. As direct injection of samples containing sulfuric acid may cause damage in the column, a liquid–liquid extraction step was used to prevent such damage [15, 16]. In order to rule out the influence of methanol and ethanol on derivatization, we first analyzed their concentrations before derivatization. Even if other derivatization agents (like isopropanol) are used [9], it is possible to have the same effect in cases of methanol and ethanol presence in patient samples; then the reported final concentration would be false. Therefore, it is better that methanol and ethanol concentrations are detected before derivatization.

According to methanol kinetics and the late occurrence of methanol toxicity symptoms, there is a time interval between consumption and death [1, 8, 25]. Variations in dose, genetic factors, physical conditions like weight and sex, and history of alcohol consumption affect the metabolism of methanol; they also have an impact on the severity of its toxicity, and the time gap between its consumption and death [3, 5, 25]. Hence, information regarding corpse history can be useful for detecting the cause of death. However, since we collected samples from the Department of Forensic Medicine, the complete detailed information was not available as the family of the deceased, in most cases, refused to answer such questions. Generally, in this study, the dose, elapsed time, concurrent use of other substances, and history of the poisoned person remained unknown. This was the most important limitation of this work. Consequently, it was very difficult to correlate the observed methanol concentrations to the incidence of death.

Following the investigation of relationships between the observed concentrations, we found a strong correlation between methanol concentration in the blood and vitreous samples, similar to the finding of Graham R. Jones et al. [22]. Moreover, like the mentioned study, we found poor correlations between methanol and formic acid concentrations in both the blood and vitreous samples. Also, there was a weak correlation between the vitreous and blood concentrations of formic acid [22]. This can happen due to different circumstances in the metabolism of methanol and formic acid production including genetics, simultaneous use of other substances and dosage, and received medical treatment [25]. These issues can explain the difference between formic acid formation and methanol metabolism. Since there was a weak negative correlation between the blood concentrations of methanol and formic acid in our study, formic acid concentration increased alongside the decrease of methanol blood concentration.

Cytochrome oxidase was inhibited by format in concentrations of 5–30 mmol/L and/or above 20 mg/dl [1]. In previous reports, poisoning and death happened following a wide range of methanol doses [12]. According to numerous studies in this field, there are considerable differences between individuals and reports stating concentrations that may cause poisoning and death [12]. In the study by Graham R. Jones et al. [22], it was shown that the majority of vitreous and blood samples from postmortems had formic acid in concentrations around 0.5 g/L or above. They also stated that the lowest methanol concentration in blood associated with death was 70 mg/dl. In the present study, lower concentrations associated with death were observed. This was due to different genetic conditions in metabolism, which resulted in different concentrations. In cases involving the simultaneous detection of ethanol (more than 20 μg/dl) and methanol or formic acid, poisoning by contaminated beverages was confirmed. With regard to this, in cases of methanol or formic acid detection (without ethanol observation), only poisoning by methanol was confirmed.

## Conclusion

In this study, we could confirm methanol poisoning by the determination of formic acid in each sample (vitreous or blood), even in cases where there was a considerable time gap between the use of methanol and death (despite methanol negative); but we did not find a new relationship between concentrations. Also, in cases with high concentration of formic acid, the cause of death was confirmed. Therefore, using this method for the measurement of formic acid, the degree of toxicity and the cause of death can be estimated.

## Abbreviations

FID: Flame Ionization Detector
Formate: Formic acid
GC/MS: Gas Chromatography with Mass Detector
HSGC: Headspace Gas Chromatography
